# Access to Healthcare for Children and Adolescents with a Chronic Health Condition during the COVID-19 Pandemic: First Results from the KICK-COVID Study in Germany

**DOI:** 10.3390/children10010010

**Published:** 2022-12-21

**Authors:** Julia M. Göldel, Clemens Kamrath, Kirsten Minden, Susanna Wiegand, Stefanie Lanzinger, Claudia Sengler, Susann Weihrauch-Blüher, Reinhard W. Holl, Sascha R. Tittel, Petra Warschburger

**Affiliations:** 1 Department of Psychology, Counseling Psychology, University of Potsdam, 14476 Potsdam, Germany; 2 Center of Child and Adolescent Medicine, Division of Pediatric Endocrinology and Diabetology, Justus-Liebig-University, 35392 Giessen, Germany; 3 Program Area Epidemiology, Deutsches Rheuma-Forschungszentrum (DRFZ), 10117 Berlin, Germany; 4 Department of Pediatric Respiratory Medicine, Immunology and Critical Care Medicine, Charité-Universitätsmedizin Berlin, 10117 Berlin, Germany; 5 SPZ-Pädiatrische Endokrinologie und Diabetologie, Charité-Universitätsmedizin Berlin, 13353 Berlin, Germany; 6 Institute of Epidemiology and Medical Biometry, ZIBMT, Ulm University, 89081 Ulm, Germany; 7 Germany and German Center for Diabetes Research (DZD), 85764 Neuherberg, Germany; 8 Department of Pediatrics I, Pediatric Endocrinology, University Hospital Halle (Saale), 06120 Halle (Saale), Germany

**Keywords:** chronic health condition, children and adolescents, health care, COVID-19 pandemic, diabetes, rheumatic diseases, obesity

## Abstract

This study examines the access to healthcare for children and adolescents with three common chronic diseases (type-1 diabetes (T1D), obesity, or juvenile idiopathic arthritis (JIA)) within the 4th (Delta), 5th (Omicron), and beginning of the 6th (Omicron) wave (June 2021 until July 2022) of the COVID-19 pandemic in Germany in a cross-sectional study using three national patient registries. A paper-and-pencil questionnaire was given to parents of pediatric patients (<21 years) during the routine check-ups. The questionnaire contains self-constructed items assessing the frequency of healthcare appointments and cancellations, remote healthcare, and satisfaction with healthcare. In total, 905 parents participated in the T1D-sample, 175 in the obesity-sample, and 786 in the JIA-sample. In general, satisfaction with healthcare (scale: 0–10; 10 reflecting the highest satisfaction) was quite high (median values: T1D 10, JIA 10, obesity 8.5). The proportion of children and adolescents with canceled appointments was relatively small (T1D 14.1%, JIA 11.1%, obesity 20%), with a median of 1 missed appointment, respectively. Only a few parents (T1D 8.6%; obesity 13.1%; JIA 5%) reported obstacles regarding health services during the pandemic. To conclude, it seems that access to healthcare was largely preserved for children and adolescents with chronic health conditions during the COVID-19 pandemic in Germany.

## 1. Introduction

Healthcare systems and healthcare providers all over the world had to face multiple challenges throughout the Coronavirus disease 2019 (COVID-19) pandemic [1,2]. Initially, the main focus was on the severe acute respiratory syndrome coronavirus 2 (SARS-CoV-2), leading to changes in access to healthcare for individuals with other diseases [1,3].

Overall, the COVID-19 pandemic has led to a national [1,4,5] and international [6,7,8,9] decrease in hospital admissions and outpatient visits, including pediatric care [5,10,11,12]. Diagnosis and treatment of non-communicable diseases are among the most frequently disrupted health services (HS) [13]. However, delayed diagnoses of chronic health conditions (CCs) can lead to serious complications, i.e., diabetic ketoacidosis [14,15].

Appointments were either canceled by healthcare facilities to prioritize capacities or by patients for fear of exposure to SARS-CoV-2 [8,16,17], particularly among people with CCs [8,17]. To mitigate that problem, many institutions have implemented remote healthcare, primarily as online consultations [18,19].

While the number of hospital admissions for acute illnesses has meanwhile returned to normal [7], research yields inconsistent findings regarding medical care for patients with CCs. In a study conducted in New York, Blecker et al. [7] found a persistent decline in hospital admissions in spring 2020, whereas Du et al. [20] described steady medical care by general practitioners and a decline in specialist visits in spring 2020, followed by an increase from July 2020 in a German study.

However, individuals with a CC increasingly need continuous and comprehensive medical and psychosocial care [21,22]. A guaranteed supply of medication is necessary, along with regular check-ups and further assistance. These regular visits decreased particularly during the COVID-19 pandemic [23,24]. Moreover, anxiety about medication shortages and problems regarding communication with and between healthcare providers were reported [1].

Although first studies are available examining the access to healthcare for patients with and without CCs [1,20,23,24], there is not yet sufficient evidence on utilization and self-assessed quality of healthcare for afflicted children and adolescents during the COVID-19 pandemic. Therefore, this study examined exploratorily how healthcare for children and adolescents with CCs was provided during the Delta and Omicron waves of the COVID-19 pandemic (2021/2022) and how satisfied parents were with it. We focused on three exemplary CCs, namely type 1 diabetes (T1D), obesity, and juvenile idiopathic arthritis (JIA), which represent common clinical pictures with increased burden for afflicted families and diverse demands for healthcare facilities.

## 2. Materials and Methods

### 2.1. Study Design and Procedure

Data are part of the KICK-COVID study, a prospective observational study in Germany to examine the influence of the COVID-19 pandemic on the wellbeing of underage children and adolescents with CCs and their families [25]. This study implements a mixed-methods-approach combining quantitative and qualitative items. Data were collected between June 2021 and July 2022, corresponding to the interim period, the 4th (variant of concern (VOC) Delta), the 5th (VOC Omicron) and the beginning of the 6th (VOC Omicron) wave of the COVID-19 pandemic in Germany [26]. Recruitment and data collection was conducted via three established pediatric patient registries: the German Diabetes Prospective Follow-up Registry (DPV) [27], the German Obesity Prospective Follow-up Registry (APV) [28], and the National Pediatric Rheumatology Database (NPRD) [29]. Within these registries, comprehensive health data are routinely collected from all participating children, adolescents, and parents.

Participating clinical facilities from the registries were asked to forward the paper-and-pencil questionnaires to parents of their pediatric patients (<21 years) during the child’s and adolescent’s routine checkups. Electronic data input was carried out by the clinical facilities (DPV, APV) or the registry (NPRD).

The study was conducted following the Declaration of Helsinki as well as the EU General Data Protection Regulation (GDPR). The ethics committee of the University of Potsdam approved the KICK-COVID study. The ethics committee of Ulm University approved the analysis of anonymized data from the DPV and APV; the NPRD was approved by the Charité-Universitätsmedizin Berlin. Participants’ consent to processing their clinical data was given as part of the initial enrollment for the respective registry. The Study was preregistered at the German Clinical Trials Register (DRKS; www.drks.de, accessed on 20 December 2022; no. DRKS00027974).

### 2.2. Measures

Sociodemographic and disease-related data including postal codes or rather districts and data on disease severity were provided by the patient registries. The healthcare situation was assessed via 10 self-constructed items, designed by experts in CCs, and answered by the parents.

#### 2.2.1. Visits and Cancellation of Appointments

The frequency of previous disease-specific consultations in the past 12 months was assessed by one item (“How often have you been to the disease-specific consultation with your child in the last 12 months?”) with seven answer categories (“not at all”/“1×”/…/“6× or more”). Additionally, participants indicated whether consultations had been canceled due to the COVID-19 pandemic in the last 12 months (“yes”/“no”). Further items assessed the number of canceled appointments, whether cancellations were initiated by the healthcare facility or by the family, and the reasons for cancellations by the families (fear of infection, corona-related increased organizational effort, or other reasons recorded in an open answer).

#### 2.2.2. Telemedical Care

The availability of remote healthcare offers (e.g., video consultation) was assessed by one item (“yes”/“no”). Parents rated their satisfaction with those services on an 11-point numerical rating scale (NRS, 0 = “very bad” to 10 = “very good”).

#### 2.2.3. Cancellation of Further Multi-Professional Appointments

Cancellation of further multi-professional appointments in the last 6 months was assessed with one item (“How many non-medical treatments have been canceled due to the Corona pandemic in the last 6 months?”-“none”/“<25%”/“< 50%”/“<75%”/“all”/“does not apply”). Items referred to individual training, group training, and psychological support (T1D, obesity) or physiotherapy, occupational therapy, and psychological support (JIA).

#### 2.2.4. Satisfaction with Healthcare

Overall satisfaction with disease-specific care over the past 12 months was assessed via an 11-point NRS (0 = “not at all satisfied” to 10 = “very satisfied”), and an open question asking for two different aspects (“What did not work for you? Did you miss any further support?”).

### 2.3. Statistical Analyses

Due to differences in the registries’ organizational framework, data analysis was carried out separately for each CC.

To check the representativeness of the ad hoc samples, the proportion of responders among the possible responders was calculated. Possible responders are understood to be those families who take part in the respective registry, who had an appointment at one of the participating facilities during the relevant period and whose data were available by a corresponding date. Univariate and multivariate logistic regression were performed to determine the effect of child’s or adolescent’s sex, age, disease severity and disease duration (only for T1D, JIA) on participation in the study.

Statistical analysis proceeded mainly on a descriptive level. Unadjusted outcomes were presented as median with interquartile range (IQR) or as percentage (%). Spearman correlations were calculated for each ordinal and interval scaled item with child’s age, disease severity and duration (T1D, JIA), socioeconomic deprivation, and extent of COVID-19-related restrictions at the time of study participation. Only significant correlations are reported.

Disease severity was recorded via hemoglobin A_1C_ (HbA_1C_) value (mmol/mol [%]) in the T1D-sample, via Body Mass Index–Standard Deviation Scores (BMI-SDS) in the obesity-sample, and in the JIA-sample via disease activity assessed by the doctors’ estimation on a 21-point NRS [30]. The German Index of Socioeconomic Deprivation [31] assessed socioeconomic deprivation using postal codes (T1D, obesity) or districts (JIA). Low, medium, and high socioeconomic deprivation were grouped by terciles. The Oxford COVID-19 Government Response Tracker [32] was used to account for the time-specific extent of COVID-19-related restrictions.

Questions with an open response format were analyzed qualitatively in a two-step approach. First, a categorization system was formed based on the responses from the JIA-sample. In the second step, responses from the other two samples were assigned to the existing categories, modifying the initial category system. Assignment to categories was checked by several qualified raters, and in case of discrepancies, discussed in the group.

A two-sided *p*-value < 0.05 was considered statistically significant. Statistical analyses were performed using IBM SPSS Statistics Version 28 and SAS statistical software version 9.4 (build TS1M7).

## 3. Results

In total, 905 parents completed the questionnaires in the T1D-sample, 175 in the obesity-sample, and 786 in the JIA-sample. The proportion of responders related to possible responders was 26.0% in the T1D-sample, 11.5% in the obesity-sample and 21.2% in the JIA-sample. Univariate logistic regression demonstrated a significant prediction of disease severity on the study participation in the T1D-sample (*p* < 0.01; OR = 0.82; 95%-CI [0.78, 0.88]) with a small effect size and remained in the multivariate logistic regression. A significant univariate regression was found for age (*p* < 0.01; OR = 0.97; 95%-CI [0.96, 0.99]) and disease duration (*p* = 0.01; OR = 1.03; 95%-CI [1.01, 1.05]) in the JIA-sample. The effect sizes for age and disease duration were small and remained in a multivariate logistic regression. With respect to obesity, no significant group differences were observed.

Table 1 summarizes the characteristics of each disease-specific sample. The median HbA_1C_ in the T1D-cohort was 56.7 mmol/mol (IQR, 49.8–64.6) [7.3% (IQR, 6.7–8.1)]; regarding the JIA-sample, median disease activity was 0.5 (IQR, 0.0–2.0), and the median BMI-SDS in the obesity-sample was 2.2 (IQR, 1.9–2.6).

### 3.1. Type 1 Diabetes

The median frequency of attending disease-specific consultations in the last 12 months was 4 (IQR, 3–4; *n* = 687). Patients with a severe disease attended the consultation more often (Spearman’s ρ = 0.095, *p* = 0.01, *n* = 687). 14.1% (*n* = 98/695) of respondents reported consultation cancelations with a median incident of 1, with more appointments canceled for children of younger age (Spearman’s ρ = −0.076, *p* = 0.02, *n* = 905). Regarding the canceled appointments, 48% (*n* = 49/103) of parents reported themselves, 39% (*n* = 40/103) the healthcare provider, and 14% (*n* = 14/103) both sides as initiators. The most common reason for missed appointments were health-related concerns (46%; *n* = 43/94). Of note, COVID-19-related reasons were reported in 35% (*n* = 15) of these cases, and 65% (*n* = 28) referred to other illnesses or did not specify their health-related concerns (see Table 2).

More than half (56.6%, *n* = 250/442) of the respondents indicated that alternatives for the outpatient visits were offered. Median satisfaction with these alternatives was 10 (IQR, 8–10; *n* = 316). Significant negative correlations were observed between satisfaction with remote healthcare and disease duration (Spearman’s ρ = −0.127, *p* = 0.02, *n* = 316).

In general, most parents indicated no cancellations of individual training (92.2%, *n* = 308/334), group training (87.7%, *n* = 286/326), or psychological support (92.8%, *n* = 284/306).

Overall satisfaction with diabetes-specific care was quite high, with a median of 10 (IQR, 9–10; *n* = 677). Higher satisfaction was associated with lower disease duration (Spearman’s ρ = −0.108, *p* = 0.01, *n* = 677). Concerning the perspective on problems and possible solutions related to the healthcare situation, only 10.2% (*n* = 92/905) of the parents answered the question, and 14 of them explicitly reported that no problems occurred. Most answers (51%; *n* = 40/78) referred to requests for further HS, including multi-professional interventions, i.e., training programs (63%; *n* = 25), availability of medical devices and medication (15%; *n* = 6; e.g., “catheter not available”), and services in the context of medical care (13%; *n* = 5; e.g., “shorter waiting times”). A total of 10% (*n* = 4) criticized the remote HS (e.g., “video meeting instead of telephone consultation”). For further details, see Table 3.

### 3.2. Juvenile Idiopathic Arthritis

Parents reported a median frequency of 2 visits within the last 12 months (IQR, 1–5; *n* = 753). A significant positive correlation between the number of visits and disease severity (Spearman’s ρ = 0.125, *p* < 0.001; *n* = 732), and a significant negative correlation between the number of visits and disease duration (Spearman’s ρ = −0.124, *p* < 0.001; *n* = 729) was observed. A total of 11.1% (*n* = 82/741) of the parents reported missed appointments, with a median number of 1. A total of 57% (*n* = 47/83) initiated by the family, 33% (*n* = 27/83) by the healthcare facility, and 11% (*n* = 9/83) by both. Regarding cancellations by the families, several reasons were indicated (see Table 2), with the fear of infection most frequently reported. Health-related reasons were stated by 24% (*n* = 16/68); half of them were COVID-19-specific.

In total, 13.7% (*n* = 61/445) of parents declared that they received remote consultations and rated their satisfaction with these alternatives quite high, with a median of 10 (IQR, 8–10, *n* = 54).

Concerning multi-professional services, few appointments were missed: 83.2% (*n* = 321/386) of the parents reported no cancellations of physiotherapy, 88.1% (*n* = 208/236) of occupational therapy, and 93.4% (*n* = 198/212) of psychological support.

Average satisfaction with rheumatological care reached a median of 10 (IQR, 9–10; *n* = 643). Higher satisfaction was associated with a lower illness severity (Spearman’s ρ = −0.145, *p* < 0.001, *n* = 624), a longer JIA duration (Spearman’s ρ = 0.122, *p* = 0.002, *n* = 623), and a higher socioeconomic deprivation (Spearman’s ρ = 0.115, *p* = 0.009, *n* = 511).

Concerning problems in disease-specific healthcare during the COVID-19 pandemic, only 5.9% (*n* = 46/786) provided data (see Table 3). Most responses (41%; *n* = 22/54) referred to treatment-specific challenges. A total of 12 of them (22%) requested further HS, stating lacking or limited medical healthcare offers (42%; *n* = 5), multi-professional interventions (33%; *n* = 4), and remote healthcare (17%; *n* = 2). One answer (8%) reported a lack of medication.

### 3.3. Obesity

Concerning obesity care, parents reported in median no further obesity consultations (IQR, 0–2; *n* = 95) beyond the current appointment, while 20% (*n* = 18/91) reported cancellations in the last 12 months. The median of canceled appointments was 1, with 48% (*n* = 16/33) of the parents ascribing cancellations to family decisions, 36% (*n* = 12/33) to the facility, and 15% (*n* = 5/33) to both. Among the reasons for cancellations were health-related circumstances (57%; *n* = 16/28): COVID-19-specific aspects was given as a reason by one half (see Table 2).

About 15% (*n* = 9/59) of parents indicated that they received alternative offers to replace on-site consultations. Median satisfaction with those offers was 7 (IQR, 5–9; *n* = 49). A moderate and significant positive correlation with socioeconomic deprivation could be observed (Spearman’s ρ = 0.389, *p* = 0.04, *n* = 28).

Most parents indicated no cancellations in individual training (85%, *n* = 28/33), group training (80%, *n* = 28/35) or psychological support (93%, *n* = 27/29).

Median satisfaction with obesity-specific care was 8.5 (IQR, 7–10; *n* = 100). In total, 17% (*n* = 31/183) of the parents provided data on the open question (see Table 3). Of these, seven stated that everything was fine or that they just had their first appointment. Out of the 24 answers relating to causes for dissatisfaction, 14 related to requests for further HS. Here, a lack or reduced number of medical offers (43%; *n* = 6) and a request for broader multi-professional interventions (57%; *n* = 8) were reported.

## 4. Discussion

To the best of our knowledge, this is the first study examining the access to healthcare for children and adolescents with CCs in Germany.

Altogether, access to healthcare was largely preserved for afflicted pediatric patients throughout the COVID-19 pandemic. Furthermore, our results suggest that especially those with a more severe course of a disease (T1D, JIA), and those newly diagnosed with a CC (JIA) received the care they needed [33]. Regarding the patients with obesity, the number of appointments was quite low. This might reflect a general low rate of consultations in this sample [34], which might have been even lower during the COVID-19 pandemic. To our knowledge, there is no generalizable recommendation for the frequency of the obesity-specific consultation. Thus, lower appointment rates might reflect underlying differences in disease-specific treatments. Of note, our study does not allow a conclusion about a possible decline of consultations during the pandemic.

Overall, parents in all samples reported only a small proportion of appointment cancellations. This finding may be surprising, given the large body of evidence reporting declines in outpatient consultations [1,10]. However, it is not clear whether the number of canceled appointments in this study differs from pre-pandemic numbers. The reported reasons for cancellation, including concerns about infection, increased organizational effort, and illness suggest that appointment cancellations were at least partially related to the COVID-19 pandemic. In particular, fear of infection as a reason is recurrent in the literature [12]. While it is a plausible reason, it is required to ensure that necessary appointments are kept. Campaigns increasing parental awareness should be considered [15,35,36]. In addition, the low cancellation rate of multi-professional appointments, especially group-training, may be surprising since a reduction in social contacts was recommended.

Evidence for the utilization of remote consultation is conflicting. For example, more than 75% of surveyed physicians reported offering remote healthcare in a global study [24], while about 13% of patients from a German study indicated the use of telemedical services [37]. In our study, less than 15% of parents in the obesity- and JIA-samples reported alternative service offers. In line with the literature [18], the situation was quite different for children with T1D. Underlying reasons for these disease-specific differences need to be examined. One reason might be the necessity of clinical examinations and laboratory tests in JIA, which may restrict remote healthcare. Where remote healthcare was offered, parents were overall highly satisfied.

Altogether, there was high satisfaction with disease-specific care. This might not be surprising, considering that only a few participants reported consultation cancelations. Moreover, this finding is consistent with evidence of good disease management in several CCs during the COVID-19 pandemic, e.g., a stable metabolic control in patients with T1D [38]. Concerning JIA, findings are diverse, but some indicate the steady disease management during the pandemic [39]. In contrast, frequency and disease severity have reportedly increased in obesity during the pandemic [40].

In line with this high satisfaction, a comparatively small percentage of participants provided answers about possible obstacles and solutions to healthcare during the pandemic. Requests for changes must be viewed considering this low response rate and scarce appointment cancellations. Moreover, it should be emphasized that the stated problems were only partly caused by the COVID-19 pandemic. Attention should be paid to continuing patient education. Support groups that allow communication with other families sharing the same situation, specific needs and concerns can be helpful [41,42]. One opportunity might be to offer digital services for patient education and psychosocial support, although evidence for those services needs future research because of mixed results [43,44,45,46,47].

The external validity of the study appears acceptable. Given the two-stage recruitment approach, the relatively small proportions of responders related to possible responders (11.5–26.0%) are to be expected. We can only speculate about the reasons for nonparticipation: For example, did the parents reject to participate or was the waiting time not long enough to complete the questionnaire? The disparity in sample sizes may be explained by differences in organizational framework of the three registries and structure of the healthcare system. The significant effects of disease severity (T1D), age and disease duration (JIA) on survey participation may limit the generalizability of our results. However, on account of the small effect sizes observed, their influence should be considered rather small.

### Strengths and Limitations

Several strengths should be mentioned. First, the mixed-methods approach combining qualitative and quantitative data allowed to capture the healthcare situation itself and identify individual reasons for missed appointments and improvement requests. Second, the recruitment via established registries generated data reflecting real-world care in Germany. This enables one to associate data of the questionnaire with selected health data from the registries. Third, the large sample size, especially for JIA and T1D. Fourth, with the inclusion of common but also diverse clinical pictures, we were able to descriptively identify specific characteristics of each disease to draw more general conclusions.

Several limitations should be also mentioned. First, the questionnaire consists of self-constructed items. It is quite short and involves retrospective data. Self-reports might not always reflect the objective situation of healthcare but the perception of afflicted families. Besides, no comparisons can be drawn with the pre-pandemic situation. Second, due to the conditional structure of the questionnaire, the imputation of missing data is not possible. Third, the size of the obesity-sample is rather small and can only provide first cautious indications of the respective healthcare situation. Fourth, recruitment strategy created a selective sample: only families that are part of the registries, currently in specialized care, and in consultation took part in the survey. Fifth, no comparisons across the three CCs could be made and remain to be studied.

## 5. Conclusions

Overall satisfaction with disease-specific care during the COVID-19 pandemic was quite high. Only a few appointments were canceled and only a small proportion of parents mentioned obstacles and need for improvement. Therefore, the results of this study indicate guaranteed access to healthcare during the COVID-19 pandemic for children and adolescents with CCs in Germany. Nevertheless, it also gives preliminary indications on how to improve the healthcare system and access to care. The conclusions drawn in this study can be used to provide need-based care in current and future pandemics and also provide guidance for the future use of remote care in non-pandemic times.

## Figures and Tables

**Table 1:** Sample description.

Characteristic	T1D	Obesity	JIA
N	905	183	786
Relation to child, *n* (%)			
Mother	536 (76.7)	85 (82.5)	587 (78.7)
Father	157 (22.5)	11 (10.7)	155 (20.8)
Other caregivers	6 (0.9)	7 (6.8)	17 (2.3)
Sex-children, *n* (%)			
Female	416 (46.0)	90 (49.2)	553 (70.5)
Male	489 (54.0)	93 (50.8)	231 (29.5)
Age ^a^-children, median (IQR)	13 (10–16)	11 (10–15)	12 (8–15)
Age categories- children, *n* (%)			
0–6 years	112 (12.4)	18 (9.8)	130 (16.6)
7–11 years	266 (29.4)	87 (47.5)	256 (32.7)
12–20 years	527 (58.2)	78 (42.6)	398 (50.8)
Duration of disease ^a^, median (IQR)	4 (2–7)	NA	5 (2–8)
Socio-economic deprivation, *n* (%)			
Low	402 (44.4)	11 (15.3)	217 (35.1)
Middle	342 (37.8)	13 (18.1)	205 (33.1)
High	161 (17.8)	48 (66.7)	197 (31.8)

Abbreviation: IQR, interquartile range; NA, not available. Child and Children refers to children and adolescents in this table. ^a^ in years

**Table 2:** Reported reasons for cancellation of appointments.

Reason (%)	T1D	Obesity	JIA
No. with data (%)	94 (100)	28 (100)	68 (100)
Fear of infection (e.g., “risk of infection for hospitalized patients”)	17 (18)	5 (18)	26 (38)
Organizational efforts (e.g., “patient transport not possible due to driver’s illness”)	30 (32)	6 (21)	21 (31)
Health-related (e.g., “quarantine”)	43 (46)	16 (57)	16 (24)
No necessity (e.g., “no complaints”)	0 (0)	0 (0)	2 (3)
Other (e.g., “financial”)	4 (4)	1 (4)	3 (4)

**Table 3:** Reported obstacles experienced related to health services during the COVID-19 pandemic.

Remarks (%)	T1D	Obesity	JIA
No. with data (%)	78 (100)	24 (100)	54 (100)
Appointment organization (e.g., “long interval between appointments”)	8 (10)	1 (4)	14 (26)
Availability and communication (e.g., “the team is very difficult to reach by phone”)	10 (13)	0 (0)	4 (7)
Treatment-specific challenges (e.g., “holistic support for child”)	18 (23)	5 (21)	22 (41)
Request for further health services (e.g., “lack of personal contact/check-up”)	40 (51)	14 (58)	12 (22)
Other (e.g., “everything”)	2 (3)	4 (17)	2 (4)

## Data Availability

Children and young people with chronic illnesses belong to a group of people who are especially vulnerable. It is therefore not possible to provide the data.

## Supplementary Materials

The following supporting information can be downloaded at: https://www.mdpi.com/article/10.3390/children10010010/s1, Additional Contributions.
