# Supplementary material for: Access to Healthcare for Children and Adolescents with a Chronic Health Condition during the COVID-19 Pandemic: First Results from the KICK-COVID Study in Germany
Source: Children (Basel). 2022 Dec 21;10(1):10. doi: 10.3390/children10010010 (PMC9856628; doi:10.3390/children10010010)
Supplement: Supplementary file 1 [file children-10-00010-s001.zip › children-2072468-supplementary.pdf]

Additional Contributions: Participating Centers

DPV

Augsburg Uni-Kinderklinik, Bad Kösen Median Kinderklinik, Bad Mergentheim - Diabetesfachklinik, Berchtesgaden CJD, Bocholt Kinderklinik, Bonn Uni-Kinderklinik, Bremen - Kinderklinik Nord, Darmstadt Kinderklinik Prinz. Margaret, Filderstadt Kinderklinik, Freiburg Uni-Kinderklinik, Garmisch-Partenkirchen Klinikum Pädiatrie, Gießen Uni-Kinderklinik, Gummersbach Oberbergklinikum, Hanau Kinderklinik, Heidelberg Uni-Kinderklinik, Jena Uni-Kinderklinik, Kassel Klinikum Kinder- und Jugendmedizin, Landshut Kinderklink, Leipzig Uni-Kinderklinik, Marburg Uni-Kinderklinik, Neuss Lukas-Krankenhaus Kinderklinik, Rosenheim Kinderklinik, Scheidegg Prinzregent Luitpold, Schleswig Heliosklinik Kinderklinik, Schweinfurt Kinderklinik, Siegen Kinderklinik, Stade Kinderklinik, Sylt Rehaklinik, Traunstein Kinderklinik, Wesel Marienhospital Kinderklinik, Wiesbaden Helios Horst-Schmidt-Kinderkliniken, Winnenden Rems-Murr Kinderklinik

APV

Berlin Charite Kinderklinik, Bonn Universitäts-Kinderklinik, Bremen - ZABS, Halle Universitäts-Kinderklinik, Hannover Kinderklinik Bult, Leipzig - KLAKS e. V., Wien Uni-Kinderklinik, Würzburg ambulantes Schulungszentrum

NPRD

GKJR study group:

Thomas Berger, Vestische Kinder- und Jugendklinik, Rheumatologie/Immunologie, Datteln; Regine Borchers, Universitätsklinikum Augsburg, Klinik für Kinder- und Jugendliche, Augsburg; Michael Borte, Städtisches Klinikum St. Georg, Klinik für Kinder- und Jugendmedizin, Leipzig; Normi Brück, Universitätsklinikum Carl Gustav Carus, Klinik und Poliklinik für Kinder- und Jugendmedizin, Dresden; Jürgen Brunner, Medizinische Universität Innsbruck, Kinder- und Jugendheilkunde, Innsbruck; Frank Dressler, Medizinische Hochschule Hannover, Kinderklinik, Hannover; Ivan Foeldvari, Hamburger Zentrum für Kinder- und Jugendrheumatologie, Schwerpunktpraxis am Klinikum Eilbek, Hamburg; Dirk Föll, Universitätsklinik Münster, Klinik für Pädiatrische Rheumatologie und Immunologie, Münster; Tobias Krickau, Universitätsklinikum Erlangen, Kinder- und Jugendklinik, Erlangen; Jürgen Grulich-Henn, Universitätsklinikum Heidelberg, Zentrum für Kinder- und Jugendmedizin - Kinderheilkunde I, Heidelberg; Johannes-Peter Haas, Deutsches Zentrum für Kinder- und Jugendrheumatologie, Garmisch-Partenkirchen; Maria Haller, Kinderarztpraxis, Gundelfingen; Georg Heubner, Städtisches Klinikum Dresden-Neustadt, Klinik für Kinder- und Jugendmedizin, Dresden; Nadja Hofmann, Sozialstiftung Bamberg, Klinik für Kinder und Jugendliche, Bamberg; Annette Holl-Wieden, Universitätsklinikum Würzburg, Kinderklinik und Poliklinik, Würzburg; Gerd Horneff, Asklepios Kinderklinik Sankt Augustin, Zentrum für Allgemeine Pädiatrie und Neonatologie, Sankt Augustin; Regina Hühn, Martin-Luther-Universität Halle-Wittenberg, Halle (Saale); Ales Janda, Universitätsklinikum Ulm, Klinik für Kinder- und Jugendmedizin, Ulm; Annette Jansson, Dr. von Haunersches Kinderspital der LMU, Kinderklinik und Kinderpoliklinik, München; Tilmann Kallinich, Universitätsmedizin Berlin - Charité, Klinik für Pädiatrie mit SP Pneumologie, Immunologie und Intensivmedizin Berlin; Thomas Keller, Josefinum Krankenhaus, Klinik für Kinder und Jugendliche, Augsburg; Christian Klemann, Universitätsklinik und Poliklinik für Kinder und Jugendliche, Rheumaambulanz, Leipzig; Hans Kössel, Klinikum Westbrandenburg, Kinder- und Jugendmedizin, Brandenburg; Georg Leipold, Gemeinschaftspraxis Kinder- und Jugendärzte, Regensburg; Jan Maier, Kinderarztpraxis, Leinfelden-Echterdingen; Almut Meyer-Bahlburg, Universitätsmedizin Greifswald, Abt. Allgemeine Pädiatrie, Greifswald; Kirsten Mönkemöller, Kinderkrankenhaus der Stadt Köln, Kinder- und Jugendmedizin, Köln; Tim Niehues, Helios Klinikum Krefeld, Pädiatrische Institutsambulanz, Krefeld; Nils Onken, Kinderarztpraxis, Lüneburg; Prassad Oommen, Med. Einrichtungen der Heinrich-Heine-Universität, Zentrum für Kinder -und Jugendmedizin, Düsseldorf; Bernd-Ulrich Keck, Diakonie-Klinikum Schwäbisch Hall, Kinderklinik, Schwäbisch Hall; Jürgen Quietzsch, DRK Krankenhaus Lichtenstein, Klinik für Kinder- und Jugendmedizin, Lichtenstein; Christoph Rietschel, Clementine Kinderhospital, Klinik für Kinder- und Jugendmedizin, Frankfurt; Betina Rogalski, Knappschaftsklinikum Saar Püttlingen, Abt. Rheumatologie, Sektion Kinder- und Jugendrheumatologie, Püttlingen; Michael Rühlmann, Kinderarztpraxis, Göttingen; Peggy Rühmer, Helios Vogtland-Klinikum Plauen, Fachambulanz der Klinik für Kinder- und Jugendmedizin, Plauen; Axel Sauerbrey, Helios Klinikum Erfurt, Klinik für Kinder- und Jugendmedizin, Erfurt; Fabian Speth, Universitätsklinik Eppendorf, Klinik und Poliklinik für Kinder- und Jugendmedizin, Hamburg; Klaus Tenbrock, Universitätsklinikum Aachen Klinik für Kinder- und Jugendmedizin, Aachen; Ralf Trauzeddel, Helios Klinikum Berlin-Buch, Klinik für Kinder- und Jugendmedizin, Berlin; Philipp von Bismarck, Universitätsklinikum Schleswig Holstein - Campus Kiel, Klinik für Kinder- und Jugendmedizin, Kiel; Frank Weller-Heinemann,

Klinikum Bremen Mitte – Eltern-Kind-Zentrum Professor-Hess-Kinderklinik, Zentrum für Kinder- und Jugendrheumatologie, Bremen; Daniel Windschall, St. Josef-Stift Sendenhorst, Abt. Kinder- und Jugendrheumatologie, Sendenhorst
